# Retrospective on Exploring MXene-Based Nanomaterials: Photocatalytic Applications

**DOI:** 10.3390/molecules28062495

**Published:** 2023-03-09

**Authors:** Syed Irfan, Sadaf Bashir Khan, Muhammad Aizaz Ud Din, Fan Dong, Deliang Chen

**Affiliations:** 1 School of Materials Science and Engineering, Dongguan University of Technology, Dongguan 523808, China; syedirfan@dgut.edu.cn; 2 Yangtze Delta Region Institute (Huzhou), University of Electronic Science and Technology of China, Huzhou 313000, China; 3 Dongguan Institute of Science and Technology Innovation, Dongguan University of Technology, Dongguan 523808, China; 4 School of Materials Science and Engineering, Tianjin University, Tianjin 300350, China; 5 School of Materials and Energy, Southwest University, Chongqing 400715, China

**Keywords:** MXenes, MXene-based composite, 2D materials, pharmaceuticals, water treatment

## Abstract

Nanostructural two-dimensional compounds are grabbing the attention of researchers all around the world. This research is progressing quickly due to its wide range of applications in numerous industries and enormous promise for future technological breakthroughs. Growing environmental consciousness has made it vital to treat wastewater and avoid releasing hazardous substances into the environment. Rising consumer expectations have led to the emergence of new, frequently nonbiodegradable compounds. Due to their specific chemical and physical properties, MXenes have recently been identified as promising candidates. MXenes are regarded as a prospective route for environmental remediation technologies, such as photocatalysis, adsorption, and membrane separation, and as electrocatalytic sensors for pollution recognition because of their high hydrophilicity, inherent chemical nature, and robust electrochemistry. The development of catalysts based on MXene materials for the photocatalytic breakdown of pharmaceutical wastes in polluted water is critically evaluated in this study. With an emphasis on the degradation mechanism, the photocatalytic degradation of antibiotics using MXenes and MXene-based nanocomposites is explained in depth. We emphasize the significant difficulties in producing MXenes and their composites, as well as in the degradation of drugs. The successful use of MXenes in water filtration and suggestions for future study are also presented.

## 1. Introduction

A large amount of post-production wastewater enters natural streams every day globally, which has caused contamination to a crucial level [1]. Rapid global industrial development is directly linked to a growing world population, which generates a large amount of wastewater production, mainly from the chemical, pharmaceutical, and cosmetic industries. Different washing process lines produce industrial wastes with disinfectants and surfactants. The wastewater coming from industries may have many inorganic contaminants. Inorganic contaminants may contain toxic heavy metals, e.g., cobalt, mercury, arsenic, lead, and poisonous gases including NO_x_, So_x_, Co_x,_ and NH_3_. Surface-factionalized MXenes are thought to provide a way around this problem due to their outstanding electrical characteristics, which can increase charge separation effectiveness [2]. In a previous study, rhodamine B molecules exhibited exceptional adsorption and catalytic photo-degradation of Ti_3_C_2_ MXene-Co_3_O_4_ nanocomposites, which were created using an in situ solvothermal technique [3].

**Figure 1:**
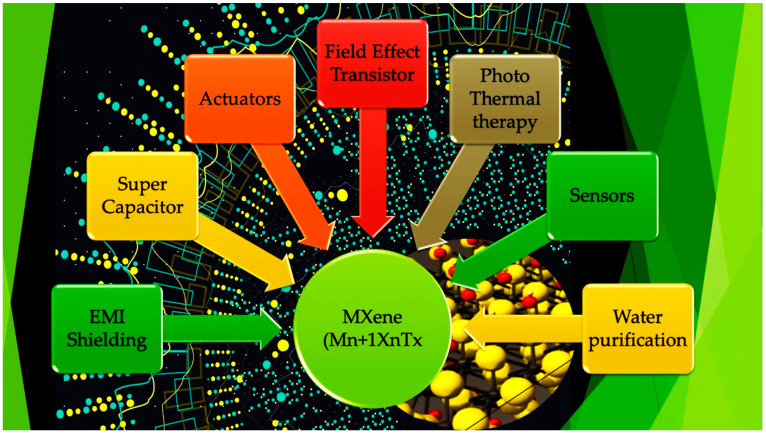
MXene applications: actuators, EMI shielding, supercapacitor, sensors, water purification, photothermal therapy, and field-effect transistor [6].

The RhB and MB dye removal rates stabilized at around 4 and 1.5 h, respectively, demonstrating the produced complex’s efficiency as a dye adsorbent. The enlarged surface area was beneficial for dye adsorption for photocatalytic needs, which could cause these characteristics. These nanocomposites demonstrated excellent photo-degradation and absorption capabilities and strong stability and reusability, as shown by an outstanding catalytic feature after eight cycle runs [3]. In another work, using an electrostatically driven method, Fang and his colleagues successfully synthesized Ag_2_WO_4_/Ti_3_C_2_ Schottky-junction photocatalyst for the first time. The Ag_2_WO_4_/Ti_3_C_2_ demonstrated an enhanced photocatalytic elimination rate for sulfadimidine (88.6%) and tetracycline hydrochloride (62.9%). The creation of the Schottky heterojunction was said to be responsible for the photocatalytic performance [4]. When 2D Bi_2_MoO_6_ was hydrothermally produced on a surface layer of nanosheets of Ti_3_C_2_ to create Bi_2_MoO_6_/Ti_3_C_2_ MXene, a more rapid photo-degradation was observed [5]. The pure Bi_2_MoO_6_ degraded only 37% of tetracycline, while the improved BT-30 photocatalyst demonstrated the highest degradation performance of tetracycline, at about 99%, within only 30 min of light irradiation. The photo-degradation of tetracycline increased with increasing Ti_3_C_2_ MXene content, as shown by the observation that the rate of reaction of BT-30 was reduced. When BT-30 degraded TC (k 0.143 min), it did so at a response rate that was 8.8 times quicker than that of pure Bi_2_MoO_6_. The improved catalyst was further examined for Cr(VI) reduction, and after 60 min, it demonstrated a 99% reduction. The different MXene applications are described in Figure 1. 

In addition to a thorough analysis of recent developments in the use of MXene-based photocatalysts for the degradation of pharmaceuticals in water, this review offers a general synthesis of the existing literature. Even though there have been several evaluations of MXenes’ ability to remove contaminants by adsorptive means, this review is focused primarily on the photocatalytic removal of antibiotics in pharmaceutical wastes. The methods used to synthesize different MXene-based catalysts, the influence of key factors on the decomposition procedure, the mechanism of decomposition, and the regeneration capacity of used catalysts are thoroughly discussed.

## 2. Introduction of Mxenes

**Figure 2:**
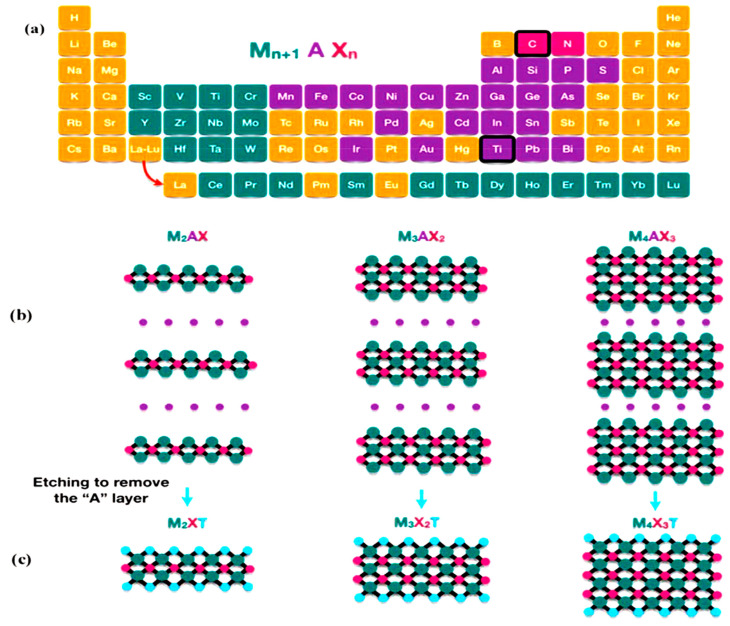
(**a**) Composition of MXene (M, A, and X represent the transition metal in green, group IIIA or IVA elements in purple, and nitrogen/carbon or both in red), (**b**) MAX-phase structures, and (**c**) MXenes [6].

Mxenes were obtained for the first time in 2011; they are a novel class of 2D transition metal nitrides, carbides, and carbonitrides [7,8,9,10,11]. Mxenes’ structures are composed of M*_n_*_+1_X*_n_* mutilated sheets having the formula of M*_n_*_+1_X*_n_*T*_x_*, where n (varies from one to four), M, X, and T_x_ symbolize the number of transition metal layers exist in the structure of MXenes, the transition metal, carbon or nitrogen, and surface terminations, respectively [12,13,14,15,16,17,18]. MXenes are generally fabricated from MAX phase (M*_n_*_+1_AX*_n_*), where A layer is typically inserted within octahedral M*_n_*_+1_X*_n_*, with a strong M/X and a comparatively weak M/A bond, as shown in Figure 2 [19,20,21,22,23]. Thus far, nearly 30 stoichiometric structures have been discovered in the MXene family, and wide-ranging exploration is in progress to grow new structures [12,24,25,26,27]. As a result of their excellent chemical and mechanical properties, Ti-based MXenes are primarily used for applications in numerous fields because of the ease of modifying their physical and chemical properties with varying MAX-phase precursors [12]. The oxygen-rich terminal groups on MXene’s surface offer active sites for chemical covalent modification, which causes the engineering of novel structures by combining suitable functional groups, therefore enhancing degradation efficiencies for the removal of organic contaminants from industrial wastewater.

**Figure 3:**
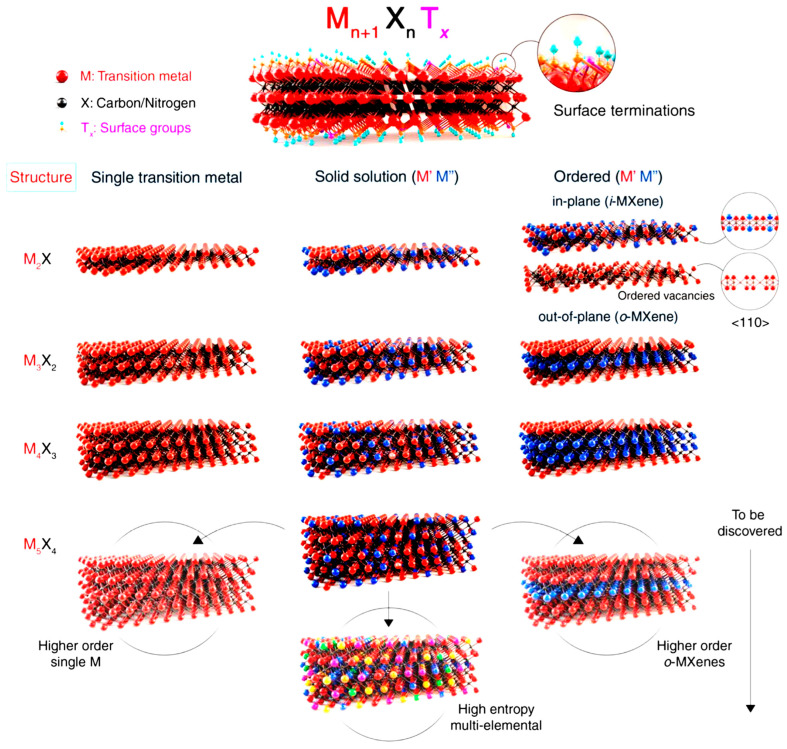
Schematic of MXenes with different n values, such as Ti_2_CT_x_ (n = 1), Ti_3_C_2_T_x_ (n = 2), Nb_4_C_3_T_x_ (n = 3), and (Mo,V)_5_C_4_T_x_ (n = 4). Reprinted with permission from Ref. [17].

A new MXene is composed of titanium carbide, which is fabricated by optimized etching of aluminum atoms in a hexagonal ternary carbide-layered structure with strong acid, ideally using hydrofluoric acid at room temperature [7]. Thus far, over 70 kinds of MAX and 30 types of MXenes have been discovered, and different kinds of MXenes with different n values are shown in Figure 3. As per theoretical calculations, there is a possibility to invent even more fascinating MXenes with exceptional properties. So far, about 30 MXene structures have been reported, which are obtained mainly by adding transition metals in the M layers [16,24,25,26,27,28]. Ti-based MXenes (Ti_2_CT_x_ and Ti_3_C_2_T_x_) are most widely studied for their green environmental applications. In addition to carbides, nitride, and carbonitrides, MXene composites have been commonly used in numerous fields [2,29,30,31,32,33,34,35,36]. The fantastic characteristics of MXenes include biocompatibility, enlarged surface area, and activated metallic hydroxide sites [20,22,37,38,39,40,41,42,43,44,45,46].

**Figure 4:**
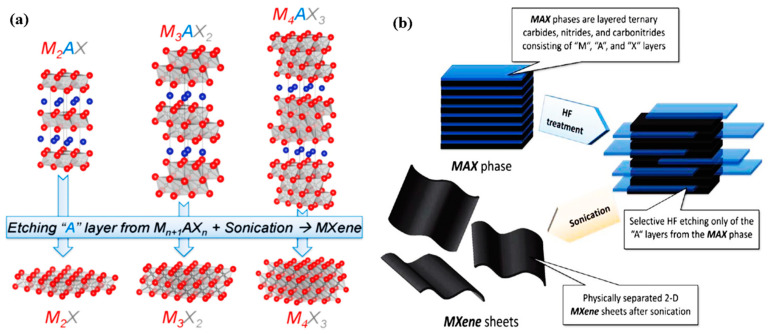
(**a**) Possible technique for fabricating of MXenes from MAX phases. Reprinted with permission from Ref. [9]. (**b**) A schematic for exfoliation route of MAX phases and production of MXenes. Reprinted with permission from Ref. [8].

MXenes can form a stable colloidal solution in water because of their large negative zeta potential [47,48]. The functionalized surface assures the hydrophilic nature of MXenes and increases the probability of bonding with several compounds. In addition, the 2D nanostructures with a rapid transmission of charges are considered with a high surface area. MXene elements are adjustable and flexible, which shows that the bandgap of MXenes can be modified [49]. Moreover, the modification and high availability of active sites on MXenes’ surface make them more attractive. These novel 2D structures can be reprocessed (furthermore, the procedure is assisted by the capability to cycle between higher and lower valence states). Numerous studies have revealed that MXenes have strong applications, for example, in ion intercalation (K^+^, Na^+^, NH_4_^+^, etc.) and catalyst immobilization. [50] The existence of titanium oxide on the surface of Ti_3_C_2_ makes it one of the most prevalent MXenes, which also serves as a significant vehicle for electron transferal during H_2_O_2_ activation [51,52,53,54]. In recent years, MXenes have also been proven to be a promising material for applications other than water purification, such as capacitive deionization, membrane separation, and adsorption [7,51,55,56,57,58,59,60,61,62]. The etching of MAX phases and exfoliation route are shown in Figure 4.

Antibacterial/antibiofouling agent materials based on MXenes have been identified as suitable photothermal materials for photothermal membrane construction. A unique Ti_3_C_2_ MXene on a cellulose membrane was recently produced by simply immersing commercially available qualitative cellulose filter paper in an MXene solution at a concentration of 1 mg mL^−1^ for 3 s at room temperature, followed by vacuum drying for 10 min at 60 °C for several cycles. The membrane showed significant light absorption efficiency of up to 94% in a wide solar spectrum range and high antibacterial efficacy of up to 99% when tested in a suspension of *E. coli* and *S. aureus* [63]. The MXene/cellulose antibiofouling membrane was innovatively designed as a solar collector and a steam generator for purifying water. The created MXene-coated cellulose membrane, which has a flat surface and can be folded into several forms, exhibits flexibility. This study described how, following photothermal water purification, translucent condensed water was produced from the bacterial suspensions of *S. aureus* and *E. coli*. The following Table 1 explained the recent literatures for removal of pharmaceutical wastes *via* MXene-based nanocomposites.

## 3. MXenes as Adsorbents

Over the past ten years, 2D nanomaterials in which MXene family have drawn more and more scientific interest for adsorption applications [84,85]. Due to their environmentally friendly qualities, MXenes and MXene-based nanocomposites have recently been found to hold potential as adsorbents for the elimination of several environmental toxins [24,42,86] due to their high chemical and structural stability, as well as hydrophilic surfaces [87,88]. Different kinds of pollutants, for example, Cr(IV), Cd(II), Pb(II), Hg(II), and Cu, have all been successfully removed using titanium carbide (Ti_3_C_2_T_x_), which has been shown to have ideal properties as an adsorbent (II) [89,90,91,92]. Adsorption is one of the best ways to get rid of heavy metal ions when biological and chemical processes are ineffective [93,94,95,96]. The adsorption of various metal ions using Ti_3_C_2_T_x_ MXene occurs through its electrostatic and chemical interactions, high surface area, hydrophilicity, and special surface functionalities [97]. The nature of the functional group available at the surface of MXenes, which is an addition from the essence of the intercalants and etchants used during the intercalation, delamination, and etching process, is linked to the capacity of the surface of MXenes to grasp an electrostatic charge [31,98]. Electrostatic interactions enable opposing charges of possible targets existing in the wastewater. By altering the pH of the solution, the surface of MXenes can be positively or negatively adjusted to adsorb charged objects [99,100].

Various radionuclides, dyes, heavy metals, and other pollutants that are the objectives in wastewater treatment could be targets of interest. Objectives for wastewater treatment include radionuclides, heavy metals, dyes, and other contaminants. The adsorptive behavior of Cr(VI), a substantial and highly toxic mutagenic heavy metal and carcinogenic water pollution, on MXenes has been studied [101]. MXene/PEI-altered sodium alginate aerogel showed a better adsorption capacity (538.97 mg g^−1^) toward the elimination of Cr(VI) and an ultrahigh adsorption capacity (3568 mg g^−1^) for Congo Red. The composite’s special adsorption capabilities are credited with the strong electrostatic behavior and the synergistic effects of intercalation and surface adsorption. This substance also demonstrated exceptional antibacterial activity against *E. coli* and *S. aureus*. The Langmuir adsorption isotherm and pseudo-second-order kinetic model are both in good agreement with the adsorption data [102]. A new imidazole MXene nanohybrid Ti_3_C_2_@IMIZ shows a strong adsorption affinity for Cr, as reported by G. Yang et al. (VI). The adsorption performance and procedure analysis demonstrate that electrostatic contact is the primary physical adsorption mechanism [103]. It has also been shown that alkalized MXenes have a considerably increased ability for adsorbing hazardous metal ions [88]. The alkalized MXenes benefit from a better photocatalytic efficiency and an improved adsorption capability due to this treatment’s increased interlayer spacing. The successful decrease in F-atoms on the MXene’s surface and the following increase in O atoms lead to an increase in OH groups, which readily adsorb small ions and molecules in the solution, as confirmed by the EDS analysis [104].

According to Zheng et al.’s analysis of the three different types of aqueous solutions that they fabricated, more OH groups led to better alkali-Ti_3_C_2_T_x_ adsorption after activation by LiOH, NaOH, and KOH. Compared with the pure Ti_3_C_2_T_x_, each composite showed rapid methylene blue (MB) elimination rates because the terminal OH groups are so good at adsorbing cationic dyes. The MXene composite NaOH-Ti_3_C_2_T_x_ showed the highest adsorption capacity for MB among these three MXene adsorbents, at 189 mg g^−1^, followed by KOH-Ti_3_C_2_T_x_ (79 mg g^−1^) and LiOH-Ti_3_C_2_T_x_ (121 mg g^−1^). Organic contaminants may contain pharmaceuticals, dyes, pesticides, and biotoxins [105,106,107,108,109]. Wastewater complexity and multitude represent a crucial need for a highly efficient wastewater treatment process. Wastewater containing a too-high concentration of substances causes negative changes in its bioc enosis and performance when introduced to a biological reactor. Pharmaceuticals primarily cover assorted organic contaminants, for example, antimicrobial agents that may have an obstinate noxious action on microorganisms, causing hefty danger to the aquatic ecosystem. They are generated from different origins, such as from industrial, hospital, and domestic sources. Even a small amount of wastewater contains a large amount of highly complex and composite organic and inorganic contaminants. Due to their futile removal in the photocatalytic process, they are commonly discovered in natural water reservoirs.

The typical concentration of pharmaceutical contaminants in wastewater ranges between 1 ng^−1^ and 600 ugL^−1^ [110,111]. Pharmaceuticals in wastewater are somehow resistant to biodegradation, and because of this, these compounds become highly contagious for aquatic life. These pharmaceutical-activated compounds sustain harmful impacts on marine life as they are resistant to microbial degradation [112,113]. Due to the versatile chemical nature of medicines, removing these pollutants from wastewater is almost impossible. Various drugs, for example, mefenamic acid, carbamazepine, and non-steroidal anti-inflammatory drugs, are poorly eliminated from pharmaceutical wastewater, with a percentage of removal efficiency lower than 40%. Similar to the above situation, antibiotics have poor removal efficiency. With a poor removal efficiency of azithromycin (45%), erythromycin (30%), and clarithromycin (43%), pharmaceutical contaminants become persistent in the wastewater treatment process [114]. The elimination of pharmaceuticals in wastewater includes procedures for coagulation, flocculation, adsorption, ozonation, and biological treatment. 

**Figure 5:**
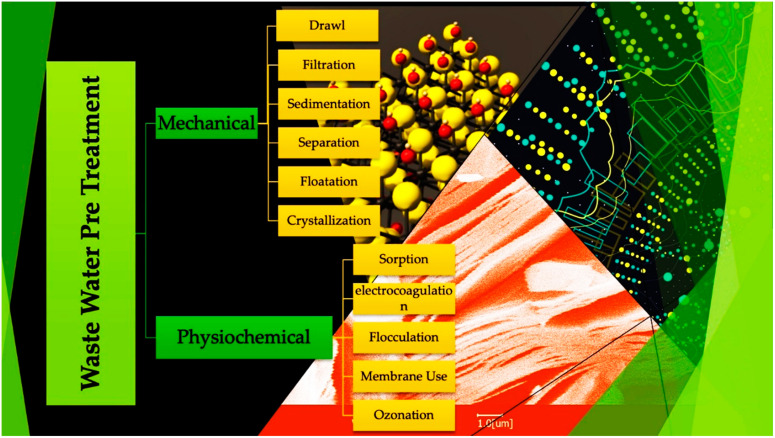
Preliminary removal methods of contaminants from wastewater [118].

The coagulation and flocculation process has been proven inefficient, with an efficiency rate of 0 to 50%. With significant equilibrium times, adsorption has been evidenced to efficiently remove pharmaceutical contaminants, with an efficiency ranging from 20 to 99% [115]. Ozonation also has good efficiency in removing pharmaceutical pollutants from wastewater treatment plants. These processes somehow cause toxic binary products during degradation, which may also cause harmful impacts on the environment [116]. Antibiotics (such as ciprofloxacin) are present in high effluents after degradation. Their non-biodegradable behavior contributes to antimicrobial resistance, which may have detrimental effects on human health. According to a global health report, antimicrobial resistance is accountable for over 23,000 casualties in the United States [117]. In recent times, photocatalysis seems to be the topic of discussion in research development as it is a renewable technology with high efficiency in eliminating antibiotics from wastewater. Photocatalytic degradation of pharmaceuticals from wastewater can resolve environmental problems with an increased ability of degradation. The following diagram represents different preliminary wastewater treatment processes (Figure 5).

In recent years, photocatalysis of pharmaceutical pollutants has attracted great attention in research due to its hopeful potential of solving energy-related and environmental issues. The photocatalytic process can be described as involving (1) the production of electrons and holes with a photocatalyst through absorbing incoming light with a sufficient amount of energy; (2) the separation and movement of photo-generated electrons and holes to the photocatalyst’s surface; and (3) the simultaneous reactions that occur (oxidation and reduction) via consuming photo-generated charge carriers [97,119,120,121]. Photocatalysis promises an effective method for the degradation of pollutants as it involves solar energy as a driving force. In recent years, nanotechnology has been widely discussed for its practical approach to eradicating organic waste products from industrial wastewater, which is attributable to their unique structural and electrochemical properties. In particular, 2D nanomaterials, which present a large surface-to-volume ratio and the thinnest structures, assures a faster processing time and a higher treatment efficiency. In recent years, 2D materials have been prominently used for the photocatalytic degradation of organic materials, which also encompasses pharmaceutical drug degradation.

## 4. Synthesis of MXene-Derived Heterostructure Photocatalyst

**Figure 6:**
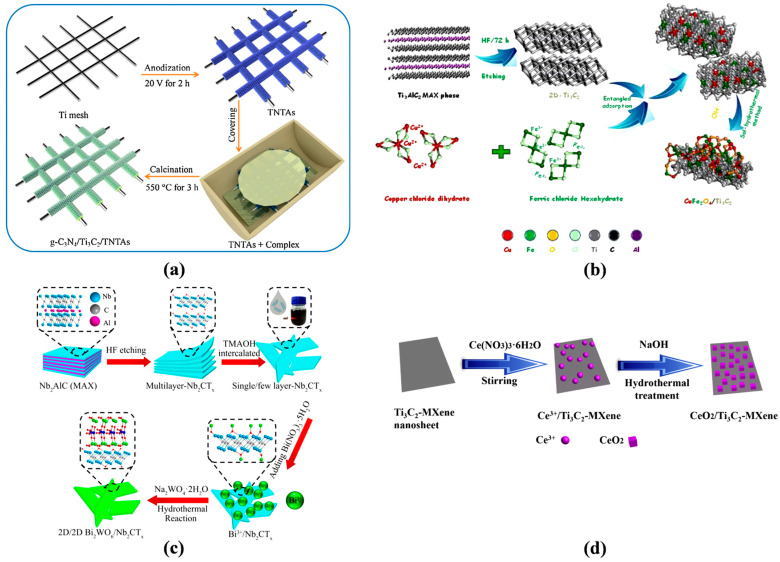
Schematic explanation of the synthesis methods of (**a**) g-C_3_N_4_/Ti_3_C_2_/TNTAs, (**b**) CFO/Ti_3_C_2_ layered heterojunctions, (**c**) Bi_2_WO_6_/Nb_2_CTxnanosheets, and (**d**) CeO_2_/MXene composites. Reprinted with permission (**a**) from Ref. [124], (**b**) from Ref. [125], (**c**) from Ref. [126], and (**d**) from Ref. [65].

In a previous study, a delaminated-Ti_3_C_2_T_x_/alkalized-C_3_N_4_ (TC-aCN) heterostructure photocatalyst was fabricated by inserting Ti_3_C_2_T_x_ into alkalized nanosheets of g-C_3_N_4_ [122]. Firstly, the delaminated Ti_3_C_2_T_x_ was synthesized by scattering the Ti_3_AlC_2_ material into a HF solution (40%), and then dispersed with continuous magnetic stirring, washed with deionized (DI) water, and dried in a vacuum furnace. In the next step, the TC-aCN was formed by dispersing the D-TC, urea, and KOH in DI water, followed by continuous stirring through the formed cyano groups. The Schottky connection between the two composites strengthen the van der Waals heterostructure, improving photo-excited electron relocation and seizing of Ti_3_C_2_T_x_. The graphitic carbon nitride (gCN) was fabricated in a calcination process based on the heterojunction of Ti_3_C_2_ and black phosphorus [123]. The synthesized materials were mixed into deionized water, and then black phosphorus nanosheets were added. The obtained solution was sonicated, vacuum dried, and then sintered for 2 h at 350 °C. Researchers have also employed anodization and chemical vapor deposition methods to synthesize photocatalysts, such as Ti_3_C_2_/g-C_3_N_4_/TiO_2_ nanotube arrays on Ti meshes [124] (Figure 6).

**Figure 7:**
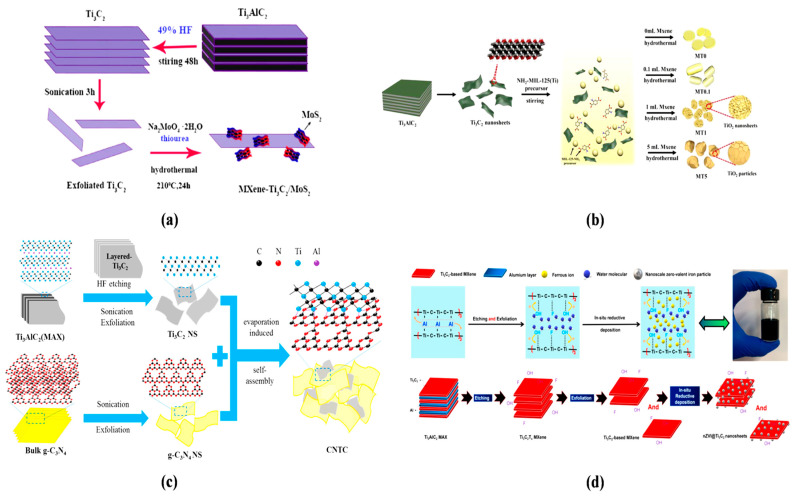
Graphic representation of the synthesis processes of (**a**) Ti_3_C_2_ and MXene-Ti_3_C_2_/MoS_2_ composites; (**b**) Ti_3_C_2_ and heterostructure of MOFs; (**c**) CNTC photocatalyst by ultrasonic dispersion using an evaporation-induced self-assembly approach and combining HF etching; and (**d**) nZVI@Ti_3_C_2_. Reprinted with permission (**a**) from Ref. [127], (**b**) from Ref. [128], copyright (2020), (**c**) from Ref. [129], and (**d**) from Ref. [130].

In one study, a homogenously distributed composite included Ti_3_C_2_, and the melamine was synthesized and implanted in the quartz boat. After that, gas was passed through the foil and enfolded the quartz boat. The photocatalyst was fabricated by sintering the quartz boat in a vacuum furnace (550 ◦C @ 3 h). The as-synthesized photocatalyst Ti_3_C_2_/g-C_3_N_4_/TNTAs revealed a higher photocurrent density compared to TNTAs (Figure 6). The CuFe_2_O_4_/MXene heterojunction was fabricated via a hydrothermal method [127]. The Ti_3_C_2_ was scattered in DI water, and then FeCl_3_•6H_2_O and CuCl_2_•2H_2_O were added. The precipitate was cleaned with DI water and dried after magnetic stirring, pH adjustment, and heating of the mixture. The MXene-Ti_3_C_2_/MoS_2_ hybrid, which has an exceptional photocatalytic efficiency, could also be synthesized using the conventional hydrothermal method, as shown in Figure 7 [127]. Similarly, Ag_2_WO_4_/Ti_3_C_2_ Schottky composite was fabricated by electrostatic traction [3].

The above figure represents the fabrication of MXene nanocomposites and nanosheets. Scientists have also discovered different processes of MXene-derived composite production.

### 4.1. Evaporation-Induced Synthesis of MXene Composites

An evaporation-induced self-assembly (EISA) technique was used to fabricate the Ti_3_C_2_/g-C_3_N_4_ composite [129]. Meanwhile, g-C_3_N_4_ nanosheets were distributed in DI water with ultrasonication, and Ti_3_C_2_ was mixed into the suspension; after that, magnetic stirring and evaporation of the solvent were performed. The suspension was then frozen and dried to obtain the Ti_3_C_2_/g-C_3_N_4_ composite. 

### 4.2. In Situ Reductive Deposition Synthesis

The researchers produced nZVI@Ti_3_C_2_-based MXene nanosheets using an in situ reductive deposition method [130]. Firstly, Ti_3_C_2_ nanosheets were mixed into a FeSO_4_ solution and ultra-sonicated. Then, the composite was moved to a flask with a continuous flow of nitrogen by slowly mixing a NaBH_4_ solution and continuously stirring, followed by reducing Fe^2+^ to Fe^0^.

Fe(H_2_O)_6_^2+^ _(aq)_ + 2BH_4_^−^ _(aq)_ → Fe^0^ _(s)_ + 2B(OH)_3 (aq)_ + 7H_2 (g)_↑

They as-synthesized heterogeneous nano-photocatalyst was made of MXene nanosheets functionalized with CoFe_2_O_4_ nanoparticles with liquid self-assembly. The MXene and CoFe_2_O_4_ nanoparticles were scattered in DI water; then, after ultrasonication, it was mixed and stirred. The as-synthesized precipitates were cleaned with DI water and ethanol. Afterward, the composite was separated, dried in an oven, and ground for advanced purposes [131].

### 4.3. The Flux Synthesis Method of Nanocomposites

Some researchers also prepared C-TiO_2_/Bi_4_NbO_8_Cl material using the flux technique. In this method, a specific quantity of BiOCl, KCl/NaCl, Nb_2_O_5,_ and Bi_2_O_3_ was mixed with Ti_3_C_2_. Afterward, the combined mixture was sintered for 1 h at 750 °C. The composite was washed with DI water and dried to achieve the C-TiO_2_/Bi_4_NbO_8_Cl [66].

### 4.4. Solvothermal Method

This method was used to fabricate Ti_3_C_2_-Bi/BiOCl heterojunction by in situ growing Bi/BiOCl on Ti_3_C_2_. Yb^3+^/Tm^3+^ co-doped Ti_3_C_2_/Ag/Ag_3_VO_4_ compounds were synthesized using the in situ reduction method via hydrothermal method [74]. The alkalized Ti_3_C_2_ was dissolved in DI water using ultra-sonication and mixed with TmCl_3_.6H_2_O and YbCl_3_.6H_2_O. The optimized amount of NH_4_VO_3_ was combined and then inserted for magnetic stirring. The resulting solution was heated hydrothermally for 8 h at 140 °C in an autoclave after adjusting the pH to 10. The final product was washed (DI and ethanol) and dried to obtain the composite material. 

## 5. Photocatalysis by MXenes

Over the past few years, photocatalysis has been widely discussed for its advanced catalytic technology for eliminating organic contaminants. The exceptional properties of MXenes, such as the band gap of MXenes that can be tuned by modifying surface chemistry, make them perfect for catalysis, thus attracting a growing interest in using MXene and its derivatives for the purification of water. MXenes display distinctive adsorptive and reductive properties with a high electronic conductivity. MXenes also own exceptional photocatalytic activity toward several organic pollutants. MXene-derived nanocomposite AgNP-loaded MXene/polymer/Fe_3_O_4_, which is fabricated by introducing dimethyl sulfoxide into MXene, reveals a high photocatalytic activity toward 2-nitroaniline and 4-nitrophenol. Working effectively under sunlight, MXenes can also be an encouraging co-catalyst for photocatalytic consequences. After the wet chemical etching method, the many bonds that have attached to MXenes make such materials a promising catalyst for photocatalysis. MXenes have admirable metallic conductivity due to the conductive metal cores in the layered structure [132,133,134,135,136,137,138]. Ti_3_C_2_T_x_ (MXene derivative) and its composites have been extensively used for wastewater treatment to eradicate several pollutants, for example, organic dyes and heavy metals. In one of the most recent studies, nanosheets of Ti_3_C_2_T_x_ with Cu_2_O composite photocatalysts with a Schottky heterojunction were created for a visible-light-induced breakdown of tetracycline (TC) antibiotics [139]. In 50 min, the composite successfully removed 97.6% of TC. The superoxide anion radical (O_2_^•^) and hole (h^+^) were responsible for the improved efficiency. Additionally, the splitting of electron and hole pair was made possible through the presence of a Schottky heterojunction. A Ti_3_C_2_-Bi/BiOCl composite was created by adding Ti_3_C_2_ nanosheets to Bi/BiOCl. The antibiotic ciprofloxacin (CIP), which is found in water, was broken down using the photoactivity of the components as they were produced. The improved CIP removal efficiency (89%) in correlation with Bi/BiOCl (71%) and BiOCl (10%) was observed [65]. Ti_3_C_2_’s capacity to create heterostructures, which boost separation efficiency, was credited with the rise in photocatalytic activity.

## 6. MXene-Based Photocatalysis

In recent years, many researchers have worked on MXene-based materials in photocatalysis. When visible light interacts with MXenes, electrons get excited and move to the conduction band (CB) of the MXene-based photocatalyst, producing holes in the valence band (VB). The holes combine with OH and make free hydroxyl radicals, whereas the electrons react with O_2_ to create superoxide radicals. The radicals are an essential part of degradation as the active species of radicals contribute to the degradation of pharmacological and organic contaminants. Consequently, non-toxic by-products are formed, i.e., H_2_O and CO_2_ [140,141]. Electrons and hole pairs are produced after irradiation (Equation (1)), where electrons are captured suddenly by the MXene sheet, thus reducing the chance of recombination (Equation (2)).

*MXene* − *composite* + *hv* → *H*^+^ + *e*^−^ (1)

*e*^−^ + *MXene* − *composite* → *e*^−^ (*MXene*
*trapping sites*) (2)

After that, the phase electrons merge with O_2_ to produce ^•^O_2_^−^, whereas the holes react with OH^−^ to create ^•^OH^−^ (Equations (3) and (4)).

*e*^−^ (*MXene trapping sites*) + *O*_2_ → *O*_2_ (3)

*h*^+^ + *H_2_O* → *OH*^−^ (4)

Both species, as represented in Equations (3) and (4), are super reactive and change pharmaceutical wastes into H_2_O and CO_2_ after degradation (Equations (5) and (6)).

OH + Pharmaceutical pollutant → CO_2_ + H_2_O (degradation byproducts) (5)

O_2_ + Pharmaceutical pollutant → CO_2_ + H_2_O (degradation byproducts) (6)

**Figure 8:**
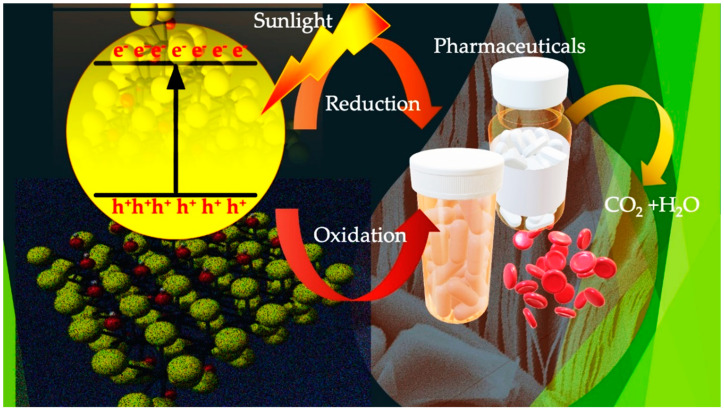
The mechanism for photocatalytic elimination of pharmaceutical wastes using MXene composite as a photocatalyst [143].

The broader absorption of photons and the quick generation of electron–hole pair mark the MXene-based photocatalyst as an enhanced photocatalytic catalyst [142]. The mechanism of photocatalytic elimination is described in Figure 8.

### MXene-Based Photocatalytic Materials

**Figure 9:**
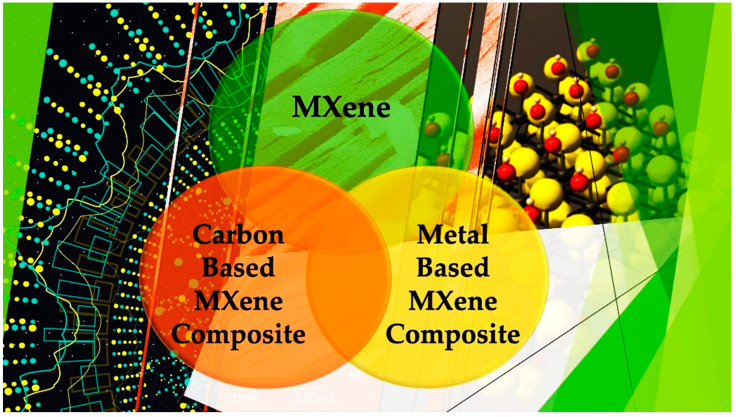
General classification of MXene-based composites. [143].

Since the discovery of MXenes in 2011, many academics have been drawn to investigate potential new prospects for photocatalysis, especially for producing materials that might be used as effective photocatalysts for CO_2_ and H_2_ reduction [31]. Additionally, such research is essential for identifying long-term responses to the world’s environmental and energy problems [144,145]. In recent years, many MXene derivatives have been studied for the degradation of pharmaceutical pollutants. Below is a description of some of the MXene-derived photocatalysts. MXenes have been coupled with carbon-based nanostructures and compounds with metal ions to provide exceptional photocatalytic properties. The general classification of MXene-based nanocomposites is shown in Figure 9.

## 7. Carbon-Based MXene Composites

Carbon-based MXene composites have been discussed by numerous scholars and have been proven as an auspicious material in visible light photocatalysis. According to the research, it may be assumed that when MXenes are combined with other chemicals, their structural makeup is altered to make them function well as photocatalysts. Fast charge carrier recombination, a wide band gap, and UV light absorption are problems for semiconductors and other photocatalytic materials. MXenes decrease other photocatalysts’ band gaps and stifle electron–hole recombination when used together. The conductive and electrical characteristics of MXenes amplify photo-generated electrons. The layered structure of MXenes offer a variety of catalytic sites as well as a surface for the pollutant’s selective adsorption. Most significantly, MXene sheets have been designed such that the bandgap of these composites makes it possible for light to be absorbed in the visible spectrum. A semiconductor with a crystalline laminar structure made up of two units, (i) 3-s-triazine ring and triazine ring (C_3_N_3_), is graphitic carbon nitride (g-C_3_N_4_) (C_6_N_7_) [146].

Nitrogen atoms’ lone-pair electrons impact the band structure [147]. Due to the weak van der Waals forces that bind neighboring layers in g-C_3_N_4_, morphological tuning is simple. Additionally, the appropriate g-bandgap of the C_3_N_4_’s structure (2.7 eV) gives it distinct optoelectronic capabilities. Due to these unique characteristics, g-C_3_N_4_ is now a preferred material for photo-degradation [148]. This improvement can be further increased by combining this advantage with 2D MXenes with a significantly higher surface area. Tetracycline hydrochloride pollutant has been successfully degraded by a visible-light active heterostructured photocatalyst g-C_3_N_4_/TiO_2_/Ti_3_C_2_. The homogenous distribution of Ti_3_C_2_ and g-C_3_N_4_ on the surface of TiO_2_ leads to enhanced charge carrier separation, resulting in an increased photocatalytic performance of up to 85.12% in 180 min. Furthermore, the stability of the photocatalyst was determined after five cycles and was found to be stable [124]. The catalyst Ti_3_C_2_/g-C_3_N_4_ has been confirmed as an efficient photocatalyst for eliminating levofloxacin antibiotics. It was discovered that Ti_3_C_2_ effectively activated g-C_3_N_4_ to increase its photo-degradation capacity. Levofloxacin’s degradation efficiency increased from 33.6% with g-C_3_N_4_ to 72% even when only 1% Ti_3_C_2_ MXene was employed. This enhanced catalyst’s capacity to respond to light photons and extend the separation of photo-generated charge carriers in the case of a composite could be because of the addition of more active sites [149]. 

A heterojunction of g-C_3_N_4_/Ti_3_C_2_ and MXene/MoSe_2_ was reported to effectively degrade enoxacin when exposed to visible light. The electron separation between Ti_3_C_2_ MXene-induced surfaces was linked to a high catalytic activity. Ti_3_C_2_ MXene served as a conduit for the flow of electrons between g-C_3_N_4_ and MoSe_2_ and served to prevent their recombination. Gatifloxacin (65%), Ofloxacin (100%), norfloxacin (80%), ciprofloxacin (80%), levofloxacin (100%), and moxifloxacin (100%) were just a few of the medications that the aforementioned composite demonstrated potential for degrading. Using MXene/g-C_3_N_4_/black phosphorus, the photocatalytic removal of ciprofloxacin has also been documented. Increased photocatalytic efficiency was built into the composite, which resulted in a synergistic interaction between MXene and g-C_3_N_4_ as well as P-bridging, which led to the production of an active heterojunction that emitted visible light. The photocatalytic degradation activity for ciprofloxacin using this photocatalyst was observed to be 99% after 60 min [123].

### 7.1. Metal-Based MXene Nanocomposites

To degrade pharmaceutical wastes, MXenes are combined with several metal-based structures, such as metal oxides, molybdates, tungstate, ferrites, sulfides, carbonates, and vanadates. Various medications have been photo-catalytically degraded using MXene–oxide composites. TiO_2_ alone has significant disadvantages, namely a high band gap and rapid electron–hole pair recombination. Using TiO_2_/Ti_3_C_2_T_x_ (MXene), the photocatalytic removal of carbamazepine has been described. The TiO_2_/Ti_3_C_2_T_x_ photocatalyst’s efficiency was improved by its modification with 2D Ti_3_C_2_T_x,_ which altered its structural restrictions, as well as by adding more e^−^ and h^+^ [150]. Different Ti_2_C-modified TiO_2_ nanocomposites had been created and embellished with Ag_2_O, Au, Ag, Pd, and PdO. These nanocomposites were discovered to be effective for refining salicylic acid, with band-gap values ranging from 0.90 to 1.31 eV. Their photocatalytic activity was ultimately enhanced, which resulted in the migration of electrons from the conduction band of TiO_2_ to Ti_2_C. Compared to pure CeO_2_, CeO_2_/Ti_3_C_2_-MXene composites produced by a hydrothermal method demonstrated 6.3 times higher photocatalytic effectiveness for tetracycline hydrochloride. It has been determined that an electric field that naturally exists between Ti_3_C_2_-MXene and CeO_2_ speeds up the synthesis of photo-induced electrons and improves the separation of charge carriers, which enhances Ti_3_C_2_-MXenes’ efficiency [65].

It has been discovered that a magnetic (Fe_3_O_4_)/Ti_3_C_2_T_x_ composite may effectively degrade diclofenac. For this case, the existence of magnetic nanoparticle Fe_3_O_4_ affects the adsorption capability, although Ti_3_C_2_T_x_ sheets provide a mechanism for the selective adsorption of organic pollutants using photocatalysis. Due to the synergistic effects of Fe_3_O_4_ and Ti_3_C_2_T_x_, this composite also demonstrated excellent stability, with just a small loss in photocatalytic capacity (8.7%) up to seven photocatalytic reaction cycles [151]. Some scientists used Ti_3_C_2_T_X_-nanosheet/Cu_2_O composite to photo-catalytically degrade tetracycline, with the photocatalytic efficiency reaching up to 97.6% in just 50 min. This outstanding photocatalytic ability was attributed to the composite’s enhanced specific surface area following its fusion with Cu_2_O. More active sites were available because of the greater surface area, facilitating a speedy electron transport to the Cu_2_O surface. As a result, the separation and migration of photo-generated charge carriers (O_2_ and h^+^) were improved, thereby improving tetracycline’s photo-degradation efficiency [139]. The increased photocatalytic degradation of carbamazepine under sunlight has been reported for the hydrothermally produced composite of Fe linked with MXene-based TiO_2_. The improved transfer of electrons produced when light is contacted results from TiO_2_ growing on the surface of MXene. Additionally, adding iron improves the composite’s photocatalytic efficacy due to a greater charge carrier separation [152].

### 7.2. MXene–Metal Tungstate Composites

Ti_3_C_2_/Ag_2_WO_4_ composite has been described in the literature for the photocatalytic degradation of sulfadimidine (88.6%) and tetracycline hydrochloride (62.9%). Ag_2_WO_4_ confined the photocatalytic capacity, but the existence of Ti_3_C_2_ increased the photocatalytic performance. The enlarged surface area and accessibility of functional groups of Ti_3_C_2_ triggered and amended photocatalysis [4]. A Ti_3_C_2_ composite with Bi_2_WO_6_ has been reported for the photo-degradation of tetracycline hydrochloride and acts as an effective photocatalyst. The photocatalytic degradation performance was pretty high, which was around 97% within 60 min of irradiation with light. The surface area of Bi_2_WO_6_ was enhanced by incorporating Ti_3_C_2,_ causing an enhancement of the adsorption capacity of Bi_2_WO_6_/Ti_3_C_2_ for tetracycline hydrochloride. The recombination rate of photo-induced charges was also decreased by the addition of Ti_3_C_2_ [68]. In this composite, the existence of MXene sheets upgraded the separation performance of photo-induced charge carriers and, finally, the photocatalytic efficiency. Compared to pure Bi_2_WO_6_, the synthesized composite (Bi_2_WO_6_/Nb_2_CT_x_) revealed an exceptional photo-degradation performance (83.1%) for tetracycline hydrochloride. Likewise, 2D heterostructures of Bi_2_WO_6_/Ti_3_C_2_ MXene have also been reported for the removal of amoxicillin within 40 min. A possible reason for the outstanding photocatalytic performance was due to the influence of Ti_3_C_2_ nanosheet for light absorption. Moreover, due to the lower Fermi level, electrons could transfer faster from Bi_2_WO_6_ to Ti_3_C_2_ [153].

### 7.3. MXene–Metal-Based Composites

A new class of materials, metal-based MXene nanocomposites have drawn a lot of interest because of their distinctive characteristics and prospective uses in a variety of industries. MXenes are a class of two-dimensional (2D) transition metal carbides and nitrides with tunable surface chemistry and high electrical conductivity. They are appealing for a variety of applications, such as energy storage, catalysis, and sensing, due to these characteristics. Metals can be included in MXene sheets to produce hybrid materials with unique characteristics. These metal-based MXene nanocomposites have undergone substantial research, and numerous applications have shown their special characteristics. For instance, it has been demonstrated that adding silver (Ag) to MXene sheets improves their antibacterial characteristics, while adding copper (Cu) increases their catalytic activity toward the reduction of 4-nitrophenol. For the manufacture of metal-based MXene nanocomposites, several techniques have been devised, including electrochemical, solid-state, and wet chemical techniques. The metal composition and final nanoparticle size may be precisely controlled using these techniques [7,154,155,156].

The composites of MXenes with ferrites, vanadates, molybdates, and phosphates have also been reported to be able to remove pharmaceutical wastes. Due to the presence of MXene in the composites, Ag_3_PO_4_/Ti_3_C_2_ showed the best photocatalytic efficiency when compared to Ag_3_PO_4_/rGO and Ag_3_PO_4_ for the photocatalytic degradation of tetracycline hydrochloride, chloramphenicol, and thiamphenicol. The photocatalytic degradation efficiency for the degradation of tetracycline did not decrease lower than 64%, even after eight cyclic runs. This shows its excellent stability due to the incorporation of Ti_3_C_2,_ which improves the catalytic performance of Ag_3_PO_4_, and also due to the introduction of several surface functional groups that are hydrophilic. As a result, a strong relationship is developed with Ag_3_PO_4_ and provides the separation of photo-generated charge carriers [70]. Cao et al. [125] fabricated CuFe_2_O_4_/MXene using the sol-hydrothermal method for the photo-degradation of sulfamethazine in visible light. CuFe_2_O_4_/MXene showed a catalytic efficiency of up to 70% for the removal of sulfamethazine due to the synergistic effects of the two different components from which it was fabricated. The enhancement in photocatalytic behavior was attributed to the transfer of photoelectrons in the synthesized composite and the prolonging of the lifetime of charge carriers due to the loading of titanium carbide. 

Cai and Zhao [5] reported visible-light responsive heterojunction Bi_2_MoO_6_/Ti_3_C_2_ for the photocatalytic degradation of tetracycline. The photocatalyst Bi_2_MoO_6_/Ti_3_C_2_ showed the highest photocatalytic efficiency and degraded tetracycline hydrochloride about 8.8 times more than the pure Bi_2_MoO_6_. A strong interaction was developed between the two interfaces, which caused a narrow distance between charge transporters and improved the separation of photo-induced charge carriers. The Bi_2_MoO_6_/Ti_3_C_2_ also produced new adsorption sites for interaction with photocatalysts and pharmaceutical wastes. A Ti_3_C_2_/Ag/Ag_3_VO_4_ composite loaded with Yb^3+^/Tm^3+^ has been reported to have exceptional photo-degradation efficiency for tetracycline. The composite eliminated about 97% of tetracycline within only 20 min, which was much higher than Ag/Ag_3_VO_4_ (68%) and Ti_3_C_2_/Ag/Ag_3_VO_4_ (83%). The presence of Ti_3_C_2_ played a significant and prominent role in modifying the photocatalytic activity of the composite. The improved photocatalytic activity of the composite was accredited to two main factors: (a) an enhancement of the absorption of visible light, and (b) a significant separation of charge carriers caused by the co-doping of Tm^3+^/Yb^3+^ metal ions. The photocatalytic mechanism showed that both species ^•^O_2_^−^ and h^+^ played a vital part in the photocatalytic elimination of tetracycline [74].

### 7.4. MXene–Metal and Oxide–Metal Nanocomposites

MXene decorated with the black phosphorous (Ti_3_C_2_T_x_/TiO_2_-BP) photocatalyst was studied and revealed to have a high-performance photo-degradation potential for tetracycline hydrochloride, which was about 92.70% under visible-light irradiation. It was confirmed that the collegial role of BP and Ti_3_C_2_T_x_/TiO_2_ intensified the photocatalytic efficiency. This synergic effect significantly enhanced the stability of BP, increased absorption capacity in visible light, enriched the photo-generated transfer of electrons, extended the photo-excited electrons’ lifetime, and postponed the recombination of electron–hole pair. Furthermore, ^•^O_2_^−^ radicals were the key active species to play a vital role in the photocatalytic procedure [157].

### 7.5. MXene–Metal Sulfide Nanocomposites

MXene–metal sulfide composites have also been studied for their photocatalytic removal efficiency for pharmaceutical wastes. For example, visible-light active CdS@Ti_3_C_2_@TiO_2_ fabricated by the hydrothermal process has been reported for its photo-degradation performance of sulfachloropyridazine, and it degraded sulfachloropyridazine completely within 88 min under visible light [149]. Moreover, Ti_3_C_2_/ZnIn_2_S_4_ has also been reported for its tremendous photocatalytic activity for the photo-degradation of tetracycline hydrochloride, which may be attributed to the advantage of 2D interaction between the two surfaces and the enlarged contact surface area for the transfer of photo-excited electrons. MXene/ZnIn_2_S_4_ was reported to have 1.8 times higher photocatalytic performance than pure ZnIn_2_S_4_ toward the breakdown of tetracycline hydrochloride. [158]. MXene/Ag_2_S composite acts as a photoactive catalyst for the removal of tetracycline hydrochloride under visible light irradiation. MXene/Ag_2_S has a much higher photocatalytic efficiency (94.91%) than either MXene (32.08%) or Ag_2_S (56.21%). The existence of several surface groups (-OH, -O, and -F) on MXene has been attributed to the increased efficiency, which improves the catalyst’s adsorption performance and catalytic capacity [159]. 

Some researchers reported a MXene-Ti_3_C_2_/MoS_2_ composite that was fabricated by the hydrothermal method to remove ranitidine under visible light. The MXene-Ti_3_C_2_/MoS_2_ composite demonstrated good photocatalytic activity against ranitidine. MoS_2_ is a photo-catalyst that is active in visible light due to its small band gap (1.8 eV). However, it has poor light absorption efficiency and performs catalytically poorly because of the quick recombination of photo-excited electrons and holes. Its interaction with Ti_3_C_2_ improves photocatalysis by facilitating the separation of charge carriers and reducing the recombination rate of photo-induced electrons and holes. [127] Furthermore, CaIn_2_S_4_/MXene Ti_3_C_2_T_x_ synthesized by the hydrothermal process has been reported for the degradation of tetracycline hydrochloride. Ti_3_C_2_T_x_ boosted the photocatalytic potential of bare CaIn_2_S_4_ by reducing charge carrier recombination and increasing absorption in the visible range (96%). Tetracycline hydrochloride was found to photodegrade with the help of superoxide radicals and holes [160].

## 8. Conclusions

The photocatalytic breakdown of antibiotics was outlined in this review. First, the effects of antibiotics in wastewater on human health and the environment were reviewed, as well as the process of antibiotic photocatalytic degradation, which depends on the production of free radicals and active oxygen species. An introduction of several popular photocatalysts, followed by an analysis of a few extensively used antibiotics, was presented. A standard method for improving a photocatalyst’s photocatalytic performance is heteroatom doping, mainly when metal atoms are utilized as dopants. The metal dopants may act as recombination centers at more significant concentrations, which can lower a photocatalyst’s effectiveness. Future studies should, therefore, concentrate on additional choices, such as doping with non-metals, such as sulfur, nitrogen, phosphorus, and boron. The efficacy of catalysts during photo-degradation investigations is also greatly influenced by physicochemical characteristics, including shape and surface areas. As has already been noted, future research on photocatalysts with different surface areas and various morphologies can significantly improve these catalysts’ activity. The degradation route offers a clear explanation of the fate and transformation of antibiotics during the process. The effectiveness of antibiotic breakdown can, therefore, be increased by investigating the photocatalytic mechanism at the atomic level. First, although the elimination rate of antibiotics is still being adjusted over the long term, the removal rate of the chemical oxygen demand during antibiotic degradation by photocatalysts is still relatively high, confirming the requirement for optimal antibiotic mineralization. A thorough investigation into intermediates is essential for enhancing the activity of catalysts because there are numerous intermediates throughout the photo-degradation process. Second, most tests involve regular and continual stirring to avoid material aggregation in the media during the photocatalytic breakdown of antibiotics, which necessitates additional energy consumption.

Thirdly, the accumulation of antibiotics in water results in the creation of microbial resistance genes, which contributes to antibiotic problems in addition to poor water quality. Research on the few resistance genes found in photocatalysts is still lacking. Finally, a photocatalyst’s capacity to be recycled is a crucial indicator of how cost-effective and practicable it is to use the photocatalyst to break down antibiotics. The design of photocatalysts with almost identical photoactivity across each cycle is desired to reduce potential waste. Additionally, it is crucial to create simpler photocatalysts that can be separated and recycled to prevent the loss of valuable materials during the photocatalytic process. Thus, from an economic perspective, it is essential to separate photocatalysts from the aqueous phase. It should be emphasized that a photocatalytic reaction’s operational costs primarily result from the photocatalyst being available for one-time usage and not being able to be recycled. In-depth research is still needed on how to use recyclable photocatalysts to responsibly degrade antibiotics.

## Figures and Tables

**Table 1:** Performance of degradation of pharmaceutical wastes from MXene nanocomposites.

Photocatalysts	Application	Optimal Degradation Efficiency	Reference
CdS–Ti_3_C_2_T_x_	HER	15.4 mmol g^−1^·h^−1^	[64]
CeO_2_/Ti_3_C_2_	Removal of C_22_H_24_N_2_O_8_ + reduction of CO_2_	80.2% in 1h	[65]
C–TiO_2_/Bi_4_NbO_8_Cl	MO, CIP, RhB, and 2&4-DCP	>95%	[66]
AgInS_2_/MXene	Reduction of N_2_	38.8 μ⋅mol g^−1^·h^−1^	[67]
Bi_2_WO_6_/Ti_3_C_2_	C_22_H_25_ClN_2_O_8_ and RhB	99.9% and 97% in 20 min and 60 min	[68]
g-C_3_N_4_/Ti_3_C_2_	HER	116.2 μ mol/h/g	[69]
Ag_3_PO_4_/Ti_3_C_2_	C_6_H_4_N_2_O_5_ and C_22_H_25_ClN_2_O_8_	>80%	[70]
TiO_2_/C_3_N_4_/Ti_3_C_2_	CO_2_ reduction	4.39 μ·mol·g^−1^·h^−1^	[71]
Ti_3_C_2_/g-C_3_N_4_	Degradation of C_5_H_5_N and C_4_H_4_S	80% in 3 h	[72]
BiOBr/TiO_2_/Ti_3_C_2_T_x_	RhB	99.8%	[73]
Ti_3_C_2_/Ag/Ag_3_VO_4_	TC, RhB, and MB	97%, 96%, and 99%	[74]
CS@g-C_3_N_4_/MX	RhB and MB	99% and 98.5%	[75]
TiO_2_@C/MXene	MB	85.7%	[76]
BPQDS/Ti_3_C_2_@TiO_2_	MO	93%	[77]
MXene@Au@CdS	HER	17,070.43 μ mol g^−1^⋅h^−1^	[78]
1T-WS_2_@TiO_2_@Ti_3_C_2_	HER	3409.8 μ mol g^−1^⋅h^−1^	[79]
Ti_3_C_2_/TiO_2_/1T-MoS_2_	HER	9738 μ mol g^−1^⋅h^−1^	[80]
TiO_2_@Ti_3_C_2_/g-C_3_N_4_	C_6_H_5_NH_2_ and RhB	99.9% and 98.2%	[81]
MXene/TiO_2_/g-C_3_N_4_	BPA, CIP, TC, and RhB	66.3%, 41.8%, 63.6%, and 92.1%	[82]
Ti_3_C_2_–Bi/BiOCl	C_17_H_18_FN_3_O_3_	89%	[83]

## Data Availability

The data will be available upon request.
